# A deep learning framework for automated detection and quantitative assessment of liver trauma

**DOI:** 10.1186/s12880-022-00759-9

**Published:** 2022-03-08

**Authors:** Negar Farzaneh, Erica B. Stein, Reza Soroushmehr, Jonathan Gryak, Kayvan Najarian

**Affiliations:** 1 Department of Computational Medicine and Bioinformatics, University of Michigan, Ann Arbor, MI 48109 USA; 2 The Max Harry Weil Institute for Critical Care Research & Innovation, University of Michigan, Ann Arbor, MI 48109 USA; 3 Department of Radiology, Michigan Medicine, Ann Arbor, MI 48109 USA; 4 Michigan Institute for Data Science (MIDAS), University of Michigan, Ann Arbor, MI 48109 USA; 5 Department of Emergency Medicine, University of Michigan, Ann Arbor, MI 48109 USA; 6 Department of Electrical Engineering and Computer Science, University of Michigan, Ann Arbor, MI 48109 USA

**Keywords:** Deep learning, CT image processing, Liver trauma, Liver disruption involvement, Hematoma segmentation, Liver segmentation

## Abstract

**Background:**

Both early detection and severity assessment of liver trauma are critical for optimal triage and management of trauma patients. Current trauma protocols utilize computed tomography (CT) assessment of injuries in a subjective and qualitative (v.s. quantitative) fashion, shortcomings which could both be addressed by automated computer-aided systems that are capable of generating real-time reproducible and quantitative information. This study outlines an end-to-end pipeline to calculate the percentage of the liver parenchyma disrupted by trauma, an important component of the American Association for the Surgery of Trauma (AAST) liver injury scale, the primary tool to assess liver trauma severity at CT.

**Methods:**

This framework comprises deep convolutional neural networks that first generate initial masks of both liver parenchyma (including normal and affected liver) and regions affected by trauma using three dimensional contrast-enhanced CT scans. Next, during the post-processing step, human domain knowledge about the location and intensity distribution of liver trauma is integrated into the model to avoid false positive regions. After generating the liver parenchyma and trauma masks, the corresponding volumes are calculated. Liver parenchymal disruption is then computed as the volume of the liver parenchyma that is disrupted by trauma.

**Results:**

The proposed model was trained and validated on an internal dataset from the University of Michigan Health System (UMHS) including 77 CT scans (34 with and 43 without liver parenchymal trauma). The Dice/recall/precision coefficients of the proposed segmentation models are 96.13/96.00/96.35% and 51.21/53.20/56.76%, respectively, in segmenting liver parenchyma and liver trauma regions. In volume-based severity analysis, the proposed model yields a linear regression relation of 0.95 in estimating the percentage of liver parenchyma disrupted by trauma. The model shows an accurate performance in avoiding false positives for patients without any liver parenchymal trauma. These results indicate that the model is generalizable on patients with pre-existing liver conditions, including fatty livers and congestive hepatopathy.

**Conclusion:**

The proposed algorithms are able to accurately segment the liver and the regions affected by trauma. This pipeline demonstrates an accurate performance in estimating the percentage of liver parenchyma that is affected by trauma. Such a system can aid critical care medical personnel by providing a reproducible quantitative assessment of liver trauma as an alternative to the sometimes subjective AAST grading system that is used currently.

**Supplementary Information:**

The online version contains supplementary material available at 10.1186/s12880-022-00759-9.

## Introduction

Trauma is the primary cause of mortality for individuals younger than 46 years old and the leading cause of years of life lost in the United States [1]. Approximately 5% of all trauma admissions are attributed to liver trauma [2]. Due to its anterior location, large size, and fragile parenchyma, the liver is the most frequently injured abdominal organ involved in blunt abdominal trauma [2–5]. Early detection and severity assessment of liver trauma with adequate treatment may result in significant reduction of morbidity and mortality [6–8].

Contrast-enhanced computed tomography (CT) is considered the gold standard technique in evaluating liver trauma and monitoring its progression over time [4, 9]. The CT-driven American Association for the Surgery of Trauma (AAST) liver injury scale is the primary tool currently in use to assess the extent of the liver trauma and guide management [10, 11]. AAST is a six-point scale with grade I signifying a small subcapsular hematoma (< 10% surface area) or laceration (< 1 cm parenchymal depth) and grade IV signifying larger laceration with parenchymal disruption affecting 25–75% of either liver lobe or 1–3 liver segments (Couinaud) [12]. However, the literature suggests significant intra- and inter-observer variability when visually assessing liver injury using the AAST grading system [13, 14]. In addition to being error-prone, visual examination might be incapable of real-time accurate quantification of the size and severity of abnormalities. Novel big data analytics and computational frameworks, however, are keys to solving such problems in digital health technology [15].

One of the main CT imaging criterion in determining AAST grade is the percentage of liver parenchyma that has been disrupted by laceration or intraparenchymal hematoma [10]. This measurement is referred to as the *liver disruption involvement* (LDI) in the remainder of this paper. Both liver laceration and intraparenchymal hematoma typically present as regions of low density as compared to adjacent unaffected/normal liver parenchyma. However, the size and shape of liver parenchymal injuries vary significantly depending on the mechanism of injury and severity of the trauma [4].

The primary aim of this study is to develop a fully automated image processing and deep learning framework that provides clinicians with quantitative assessment of LDI. This framework can act as a triage tool by rapidly assessing liver injury and its severity. To this end, both the whole liver parenchyma and liver trauma regions are automatically segmented in 3D abdominopelvic CT scans. Accordingly, the percentage of liver parenchyma that is affected by trauma will be computed.

To the best of our knowledge, except for Drezin et al. [16], no published study has proposed an automated method to segment liver trauma utilizing CT scans. However, since the goal of Drezin et al. is to detect major hepatic artery injury, it focuses on more severe cases and only includes cases with visible liver trauma (no control cases with normal liver) for training and validation purposes. Thus, it takes advantage of prior knowledge that the liver is definitely traumatically injured. Regarding automated liver segmentation, there is a body of literature that has investigated this task, however, all are focused on non-traumatic livers. Those liver segmentation techniques either implement deep learning methods [17–19] or employ classical image processing techniques [20–24]. Probabilistic atlases and active shape modeling are among the most popular classical approaches for the liver segmentation task. Farzaneh et al. [20, 21] proposed a hierarchical approach based on location and customized intensity probabilistic atlases to segment the liver. Lebre et al. [22, 23] used a location probabilistic atlas in combination with shape modeling. Rafiei et al. [25] also employed a location-based probabilistic model to generate an initial segmentation, which was then refined using an adaptive region growing technique. Okada et al. [24] and Shi et al. [26] used a probabilistic atlas to generate the initial segmentation mask and then refined it using statistical shape modeling.

The proposed framework enables an objective quantitative assessment of liver trauma as opposed to the sometimes subjective AAST grading system used in current clinical practice. The output of this study can enhance real-time liver trauma diagnostics and be used as a triage tool. Moreover, it can quantitatively measure the volumetric progression or improvement of traumatic injuries at multiple time points, guiding further investigation and management [4, 27].

## Method

### Study cohort

Before the initiation of this research project, Institutional Review Board (IRB) approval (HUM00098656) was obtained. Patient informed consent was not required given that this was a retrospective investigation. This study included 77 patients presented to the UMHS Department of Radiology for CT imaging for the evaluation of abdominal blunt force trauma between 01/01/2009 and 8/30/2014, as well as those CTs ordered by the Emergency Department. In total, the 77 CT scans comprised 8072 axial CT slices. Average patient age was 41.43 years, with a range of 18–88 years. Of the 77 patients included in this investigation, 34 had evidence of liver trauma and 43 had no evidence of liver parenchymal disruption on contrast-enhanced CT.

All CT scans were acquired in the axial plane using either GE Medical Systems (LightSpeed VCT or Discovery CT750 HD models) or SIEMENS (Emotion 16 model). Trauma protocol CT scans often include both an arterial and portal venous phase to evaluate for both arterial (e.g., aortic) and solid organ injuries. Only the portal venous phase was utilized in this study as this phase is optimal for the detection of hepatic parenchymal injuries.

To generate ground truth for all 77 patients, livers were manually annotated, which meant that the margins of the liver itself were outlined. Next, any liver laceration or hematomas were manually annotated for 34 CT scans with visible liver parenchymal disruption. Each CT scan was manually annotated slice by slice to generate binary masks (i.e., ground truth) for injury and organ. All annotations were verified by a fellowship-trained abdominal radiologist with 5 years of post-training experience (EBS).

### Experimental design

**Fig. 1:**
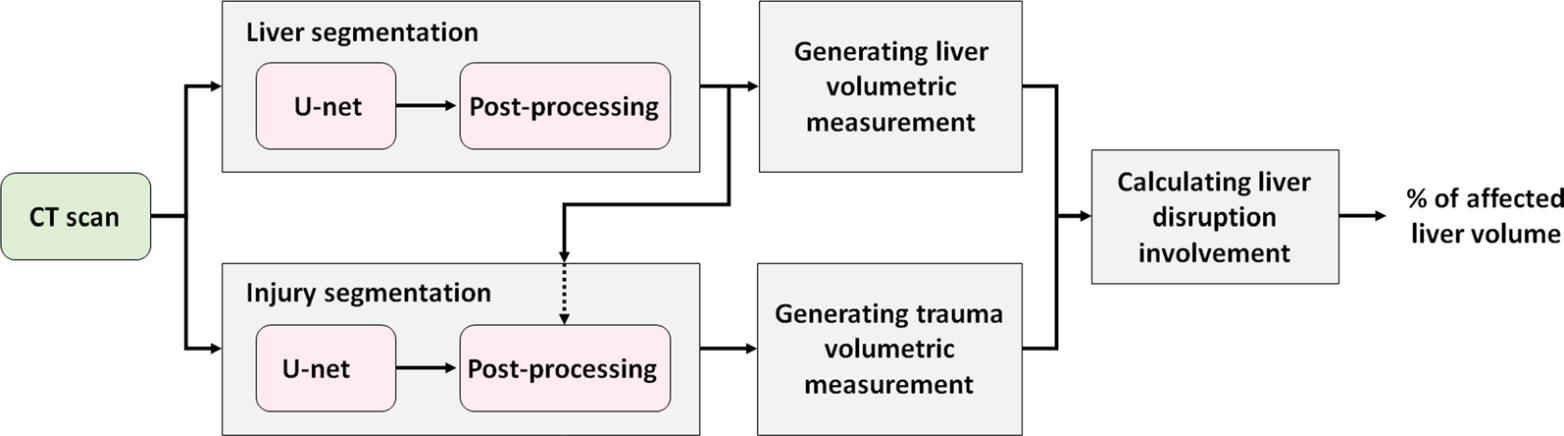
A high-level study design for liver trauma segmentation and severity assessment

The study design for liver trauma segmentation and severity assessment is shown in Fig. 1. First, deep learning-based models were developed to segment both liver organ and trauma regions. Then, to assess the severity of the liver trauma, the automatically segmented regions were processed to measure liver disruption volume and, accordingly, calculate the proportion of the liver tissue affected by those injuries (i.e., LDI).

### Liver segmentation

**Fig. 2:**
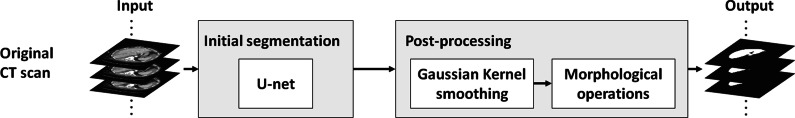
A schematic diagram of the proposed liver segmentation method

Fig. 2 demonstrates a high-level overview of the proposed liver segmentation method.

With a contrast-enhanced CT scan, we first employed a U-net model [28] to generate the initial liver mask (see Additional file 1: Method Section for the specifications of the U-net model). U-net is the most widely used deep convolutional neural networks model for biomedical image segmentation tasks and was introduced by Ronneberger et al.[28]. In the proposed model, data augmentation was performed by rotating, re-scaling, and translating the images to enhance the training dataset. Next, the post-processing module transformed the volumetric masks from the U-net model into the final segmentation map. To that end, the initial mask was filtered using 3D Gaussian kernel smoothing to achieve spatial coherency and smooth binary mask contours according to the neighboring pixels. Finally, morphological operations were used to remove small, sparse regions; fill the holes in axial planes; and exclude any region that was not connected to the largest 3D connected component.

### Liver disruption segmentation

**Fig. 3:**
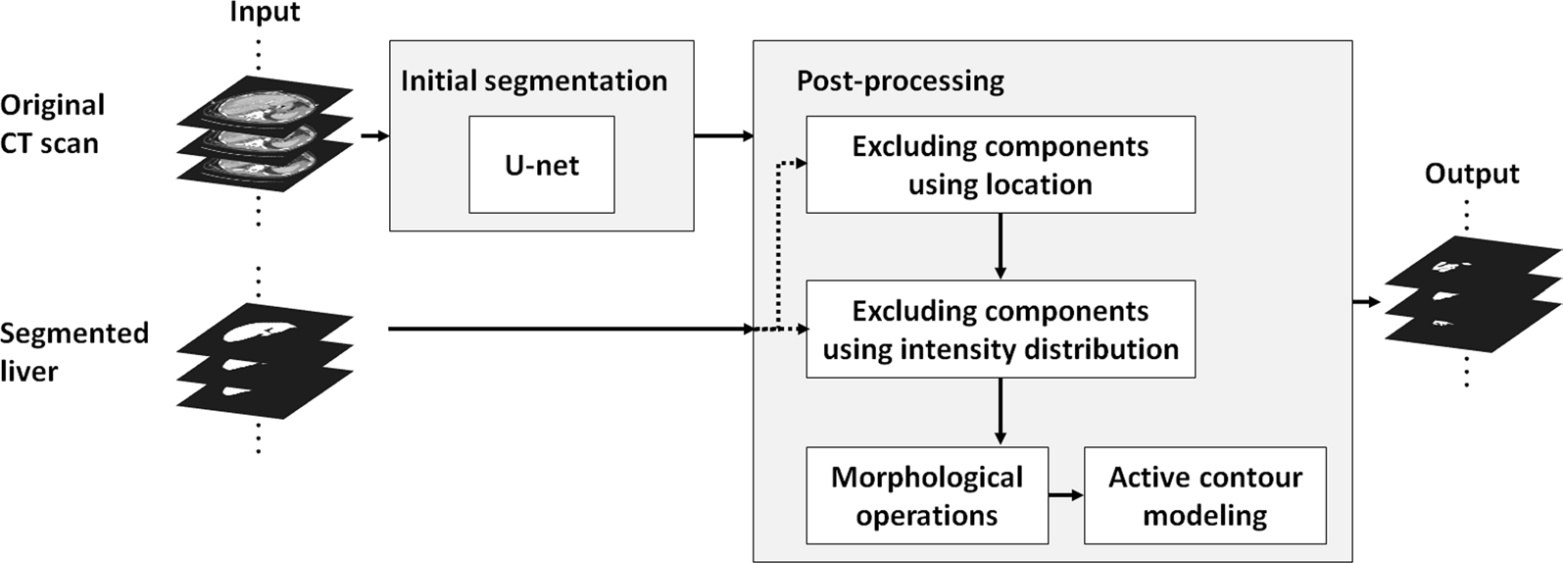
A schematic diagram of the proposed liver trauma segmentation method

As shown in Fig. 3, a second U-net backbone model was trained to segment the liver trauma regions (see Additional file 1: Method Section for the specifications of the U-net model). The post-processing module comprises the volumetric reconstruction of the U-net output, during which human domain knowledge regarding the location and intensity distribution of liver trauma was integrated into the model. It is noteworthy that the domain knowledge about location and intensity of liver trauma is incorporated into the pipeline during the model development phase. While testing the model, this information is used to automatically post-process the initial segmented region.

Considering that trauma regions are within the liver parenchyma, if more than 50% of the initial segmented trauma mask fell outside the segmented liver, the region would be excluded.

**Fig. 4:**
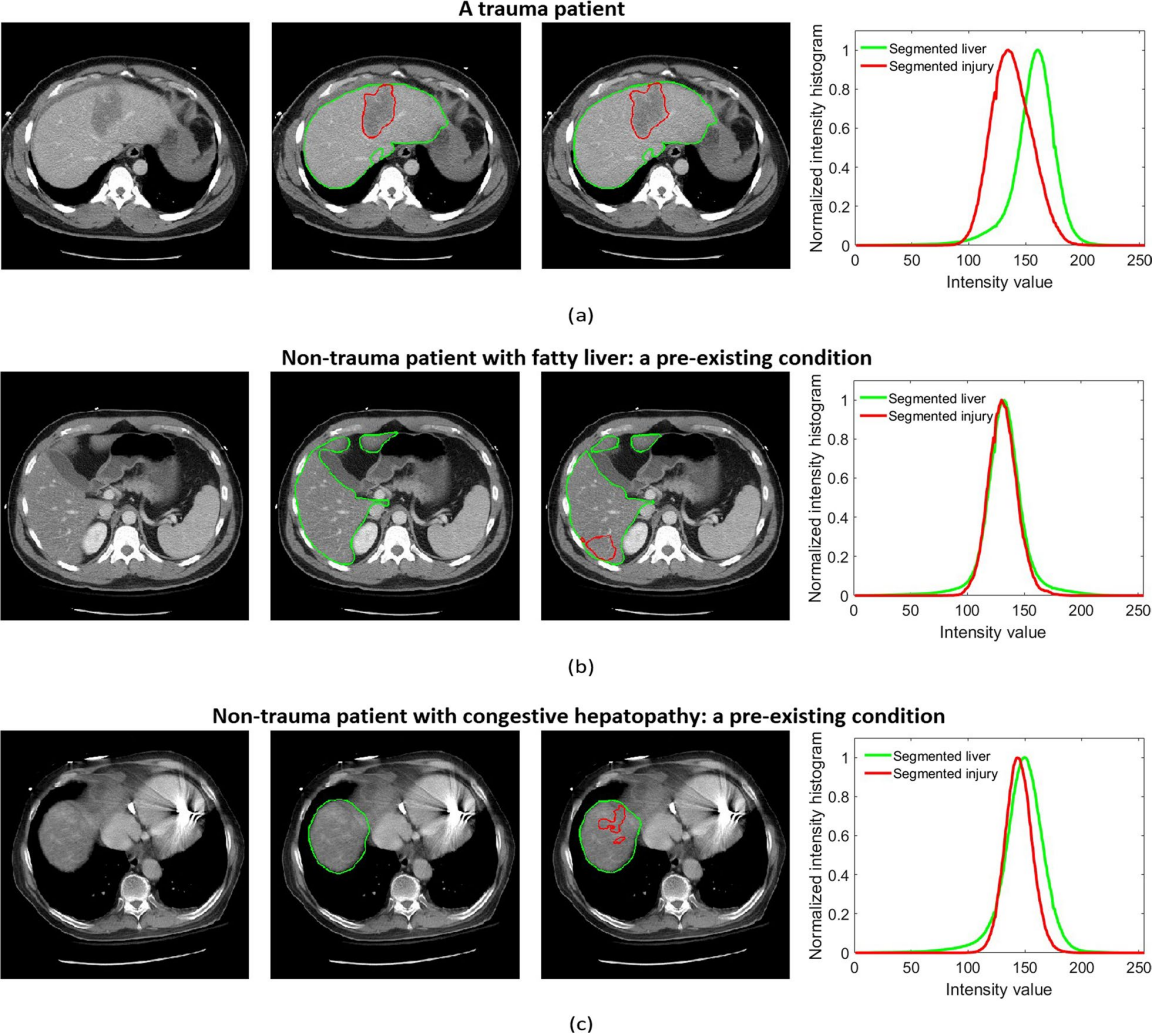
Liver trauma and organ segmentation results as well as ground truth annotations in patients with trauma or pre-existing conditions. (**a**) Axial contrast-enhanced CT image shows an intraparenchymal hematoma in the left liver lobe. (**b**) Axial contrast-enhanced CT image shows diffuse low attenuation of the right liver lobe relative to the spleen, consistent with fat deposition. This is a non-traumatic pre-existing condition. (**c**) Axial contrast-enhanced CT image shows heterogeneous enhancement of the right liver dome due to congestive hepatopathy, a non-traumatic pre-existing condition. In (**a**), (**b**), and (**c**), the first column corresponds to the original CT image; the second column corresponds to the ground truth annotations; and the third column corresponds to the automated segmentation results before post-processing. In both ground truth annotations and segmentation results, the green line shows the liver contour while the red line marks the contour of trauma regions. The fourth column compares the pixel intensity distribution inside the segmented liver and the segmented trauma region. As the -test indicated the difference between the two means of the aforementioned distributions was small for both examples of (**b**) and (**c**), the corresponding segmented injured regions were excluded during the post-processing step

Pre-existing conditions such as fatty livers (Fig. 4b) or congestive hepatopathy (Fig. 4c) lead to the different representation of non-trauma liver parenchyma on CT scans [29–31]. In theory, these pre-existing conditions could cause the U-net model to falsely detect trauma given the presence of low-attenuation of the parenchyma at baseline (Fig. 4a). To exclude regions falsely segmented as trauma (e.g., part of the normal liver parenchymal), two intensity distributions were generated, corresponding to: (1) pixels of the CT image segmented as the liver, and (2) pixels of the CT image segmented as liver trauma (Fig. 4). Next, the means of these two distributions were compared using a two- ample *t*-test. If the test statistic value was less than a fixed threshold, we concluded that the two intensity distributions were from the same texture and thus the segmented trauma region was part of the non-trauma liver parenchyma. Correspondingly, these false positive components were excluded from the segmentation using the intensity distribution.

Next, the 3D Chan-Vese active contour model (ACM) [32] was used to iteratively evolve the boundary of the initial segmentation according to local intensity and spatial coherence. The energy function *F* (*s*_1_*, s*_2_*, S*) was defined as

1 $$\begin{aligned} F(s_{1} ,s_{2} ,S) & = \mu \cdot A(S) + \nu \cdot V(S) \\ & + \lambda_1 \int_{\mathrm{inside}(S)\ } |I(x,y,z) - s_1|^2 \,dx\,dy\,dz \\ & + \lambda_2 \int_{\mathrm{outside}(S)} |I(x,y,z) - s_2|^2 \,dx\,dy\,dz \\ \end{aligned}$$

where *S* is the current surface, and *s*_1_ and *s*_2_ respectively correspond to the average intensities inside and outside the surface *S*. *I*(*x, y, z*) denotes the intensity value of a pixel at the (*x, y, z*) coordinate. Moreover, *A*(*.*) and *V* (*.*) calculate the area and volume of a surface respectively. In Eq. (1), parameters *µ*, *v*, *λ*_1_, and *λ*_2_ are constants. Following the Chan-Vese paper, parameters *λ*_1_ and *λ*_2_ were set to 1. The parameter *µ*, which specifies the degree of smoothness of the segmented region, was set to 0.1 based on prior work on an independent medical image processing problem [33]. Finally, the parameter *v* controls contraction bias, which specifies the tendency of the active contour to grow outward. This parameter was determined using a grid search. To evolve the contour, at each iteration a Sparse-Field level-set method, similar to the one proposed in Whitaker et al. [34] was implemented. After each iteration, the mask was modified to exclude the added pixels that fell outside the automatically segmented liver. Finally, morphological operations were applied to remove small, sparse regions and fill the holes in the axial plane. The effect of the post-processing step is analyzed in the result section.

### Liver disruption involvement measurement

After segmenting both liver and trauma regions (when present) as binary masks, the volumes were estimated according to respective pixel maps and the unit pixel volumes (i.e., number of pixels from the binary mask × unit pixel volume). The unit pixel volume was calculated using slice spacing and pixel spacing values extracted from CT scan metadata.

LDI was then estimated as

2 $${\text{LDI}}(\% ) = \frac{{\hat{V} (trauma)}}{{\hat{V} (r)}} \times 100$$

where $\hat{V}(\dot)$ corresponds to the estimated volume of a segmented region. For patients with no detected traumatic injury to the liver, the trauma region volume was set to zero.

### Statistical analysis

A comprehensive evaluation of the segmentation model’s performance was performed on the validation sets using Dice similarity coefficient, recall, precision, Relative Volume Difference (RVD), and Volumetric Overlap Error (VOE). RVD and VOE error measures were calculated according to definition from Heimann et al. [35] as

3 $$\begin{aligned} {\text{RVD}} & = \frac{|S| - |GT|}{{|GT|}} \\ {\text{VOE}} & = 1 - \frac{|S\,\cap\,GT|}{|S\,\cap\,GT|}, \\ \end{aligned}$$

where *GT* and *S* correspond to the ground truth and segmented masks, respectively, while |.| |.| computes the number of pixels in the corresponding mask.

To measure the variability in the LDI estimates, linear regression analysis was performed in which the computed and reference LDI measurements were plotted against each other. The linear regression relation between the two measures was then calculated. Moreover, to better perceive the algorithm’s agreement with the ground truth and potential systematic errors, a Bland-Altman analysis was employed [36, 37].

## Results

For this investigation, the presence and extent of liver trauma were assessed using the percentage of the liver affected by traumatic injuries. To compute the percentage of liver disruption, both liver and trauma regions were segmented using deep learning and image processing techniques.

This is a secondary study of an internal dataset from the UMHS that includes 77 patients, among whom 34 experienced trauma-related liver parenchymal disruption and 43 had no evidence of liver parenchymal disruption at imaging. To train and validate the segmentation models, patient-wise five-fold cross-validation was implemented. Folds were created to include a roughly balanced distribution of the trauma severity in terms of the reference LDI. The cross-validation folds remained the same for both liver and trauma segmentation tasks.

### Liver segmentation

The performance of the proposed liver segmentation algorithm is shown in Table 1. Our algorithm yielded mean Dice, recall, and precision values of 96.13%, 96.00%, and 96.35%, respectively, when tested on the internal UMHS dataset. In order to evaluate our segmentation model, in addition to the UMHS dataset, we used the publicly available 3DIRCAD dataset that includes 20 pathological CT scans with hepatic tumors in 75% of the cases [38]. Although the imaging parameters and underlying pathology of the internal UMHS and 3DIRCAD datasets are different, the 3DIRCAD dataset was only employed for testing purposes using the U-net trained only on the UMHS dataset. Based on Table 1, it can be concluded that the overall performance of the proposed algorithm is comparable with the state-of- the-art models even without tuning the weights of U-net, which indicates the generalizability of the proposed model on an unobserved dataset.

**Table 1:** Performance of our proposed liver segmentation approach compared with state-of-the-art methods. Numbers in parentheses are standard deviations. For the cited studies, scores are reported as presented in the original papers

Method	Dataset	Dice (%)	Recall (%)	Precision (%)	RVD (%)	VOE (%)
Proposed method	Internal UMHS Dataset (n = 77)	96.13 (1.49)	96.00 (2.83)	96.35 (2.09)	–0.30 (4.24)	7.40 (2.69)
Proposed method	3DIRCAD (n = 20)	94.64 (2.18)	95.06 (4.07)	94.38 (2.75)	0.83 (5.79)	10.10 (3.85)
Ahmad et. al [17]	Subset of 3DIRCAD (n = 5)	91.83 (1.37)	–	–	5.59 (6.49)	–
Lu et. al [18]	3DIRCAD (n = 20)	–	–	–	0.97 (3.26)	9.36 (3.34)
Christ et. al [19]	3DIRCAD (n = 20)	94.3	–	–	–1.4	10.7
Lebre et. al [22]	3DIRCAD (n = 20)	88 (3)	87(5)	89 (4)	–	–
Kavur et. al [39]	Subset of 3DIRCAD (n = 10)	92.0	–	–	6.42	–
Xi et. al [40]	LiTS (n = 70)	94.9	–	–	2.1	9.5

**Fig. 5:**
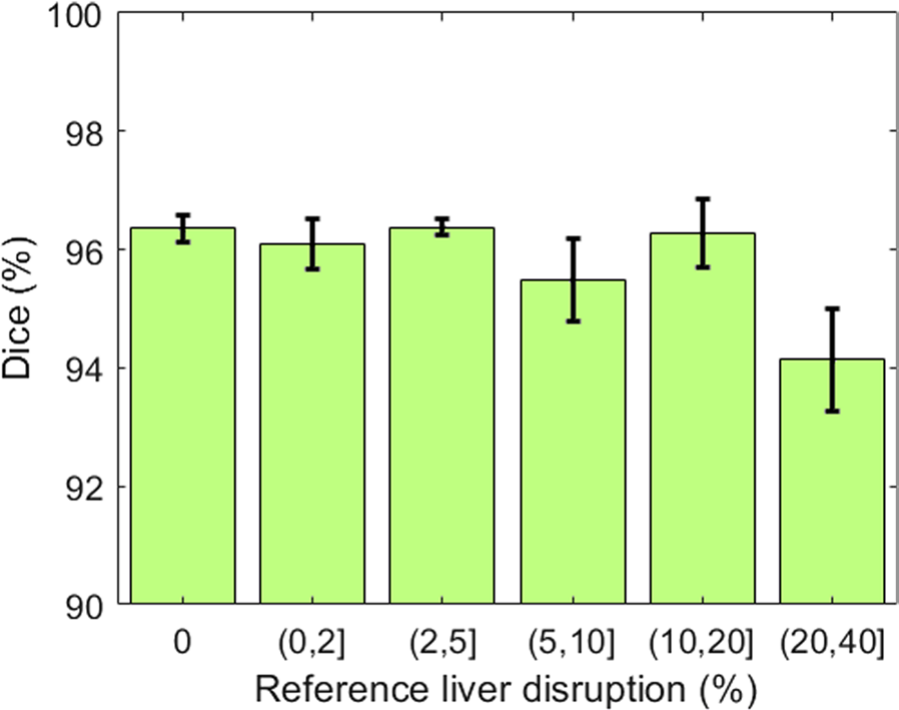
The Dice similarity coefficient for liver segmentation stratified based on LDI. Error bars represent ± 1 standard errors, the 68% confidence interval

Figure 5 shows the average Dice similarity score stratified by severity of trauma. As shown, the performance on severe cases with more than 20% of liver disruption is slightly lower as compared to smaller injuries (average Dice = 94.14%); this could be due to extensive injuries distorting the contour of the liver itself.

### Liver disruption segmentation

**Fig. 6:**
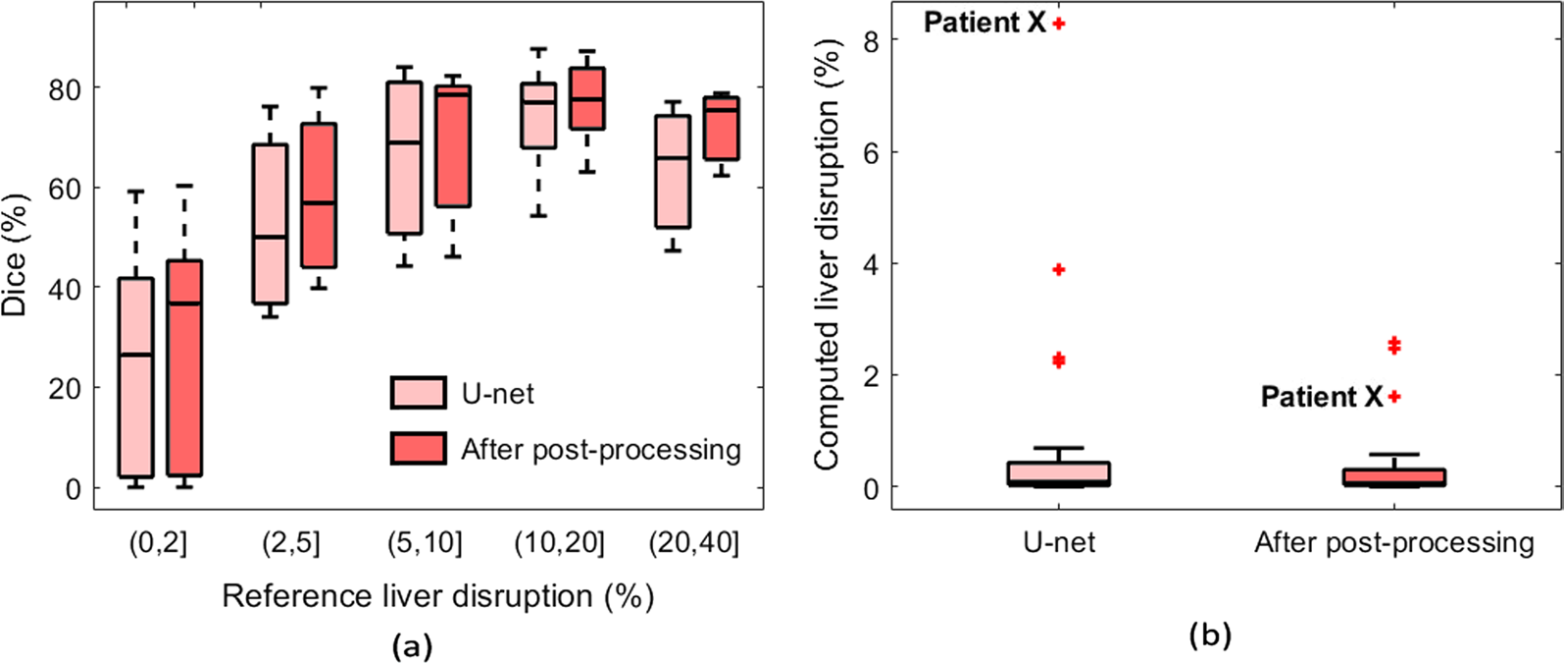
(**a**) A box plot comparing the Dice similarity coefficients of the proposed injury segmentation algorithm and U-net with respect to the percentage of liver disruption. (**b**) A box plot comparing the computed LDI for cases without liver trauma. The reference LDI is zero

Table 2 and Fig. 6a compare the liver trauma segmentation results with the ground truth. To investigate the generalizability of the algorithm with respect to injury severity, the results are stratified based on the reference LDI level. These results show that the post-processing step improves the performance in terms of the Dice similarity score. The final model achieved an average Dice of 51.21% in segmenting liver disruption while this value reached 72.45% for considerable liver disruptions that involved more than 5% of the liver. Possible etiologies for the lower performance measurements for smaller injuries with less than 2% of LDI include: (1) those subtle injuries are inherently more challenging to segment, and (2) for smaller regions, even small deviations have a greater adverse impact on the performance metrics.

**Table 2:** Performance of our proposed liver trauma segmentation approach stratified based on the severity of the injury as well as the performance of the baseline U-net architecture. Numbers in parentheses are standard deviations

Method	% Liver disruption	Dice (%)	Recall (%)	Precision (%)	RVD (%)	VOE (%)
Proposed method	0–2% (n = 15)	28.06 (23.09)	32.15 (32.90)	35.66 (27.01)	15.83 (113.41)	81.68 (16.01)
Proposed method	2–5% (n = 4)	58.35 (17.91)	54.78 (25.39)	66.21 (6.93)	− 19.16 (30.85)	57.05 (18.64)
Proposed method	> 5% (n = 15)	72.45 (11.82)	73.84 (18.60)	75.35 (11.51)	1.86 (36.03)	42.02 (13.64)
Proposed method	All (n = 34)	51.21 (27.74)	53.20 (32.56)	56.76 (27.21)	5.55 (78.88)	61.29 (24.07)
U-net (no post-processing)	All (n = 34)	47.75 (27.61)	47.32 (31.13)	56.64 (28.97)	− 2.71 (63.10)	64.50 (23.80)

Since the performance metrics including Dice, recall, and precision are not defined for the cases without any liver trauma, to evaluate the non-trauma cases, the computed LDI was used, which, ideally, should be zero for non-trauma patients. Figure 6b compares this value for non-trauma cases before and after applying the post-processing step. The average LDI for these cases is 0.27% after post-processing, with none over 2.6%. This close to zero performance shows the accuracy of the algorithm in avoiding false positives. The patient marked by “Patient X” in Fig. 6b is the patient with congestive hepatopathy, a pre-existing condition shown in Fig. 4c. Figure 4c shows the falsely segmented region before post-processing. This false positive region was excluded through the post-processing step with respect to the customized intensity distribution of the intact liver. As a result, the computed LDI of Patient X is reduced to 1.61% from 8.27%.

**Fig. 7:**
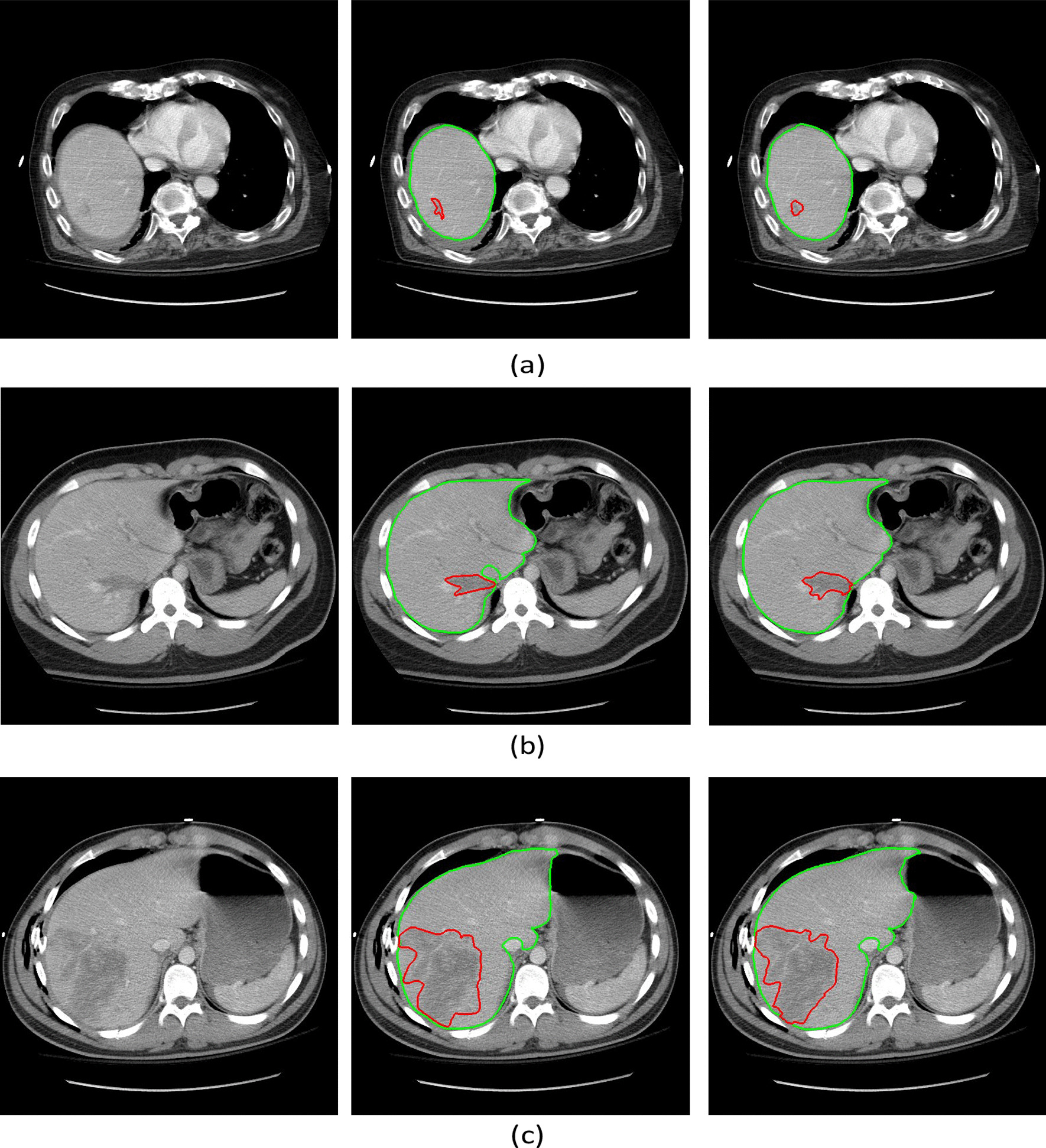
Liver trauma and organ segmentation results along with ground truth annotations in three separate patients with different levels of LDI. (**a**) Axial contrast-enhanced CT image from a patient with a very subtle 0.62% liver disruption. (**b**) Axial contrast-enhanced CT image from a patient with 2.25% reference LDI. (**c**) Axial contrast-enhanced CT image from a patient with 15.26% reference LDI. In (**a**), (**b**), and (**c**), the first column corresponds to the original CT image; the second column corresponds to the ground truth annotations; and the third column corresponds to the automated segmentation results. In both ground truth annotations and the segmentation results, the green line shows the liver contour while the red line marks the contour of trauma regions

The results of the proposed liver and trauma segmentation approaches are shown in Fig. 7. The images cover various severity levels of liver trauma. These results demonstrate that the proposed deep learning-based framework can accurately assess liver trauma, a heterogeneous clinical problem, in an automated and quantitative fashion.

### Liver disruption involvement (LDI) measurement

**Fig. 8:**
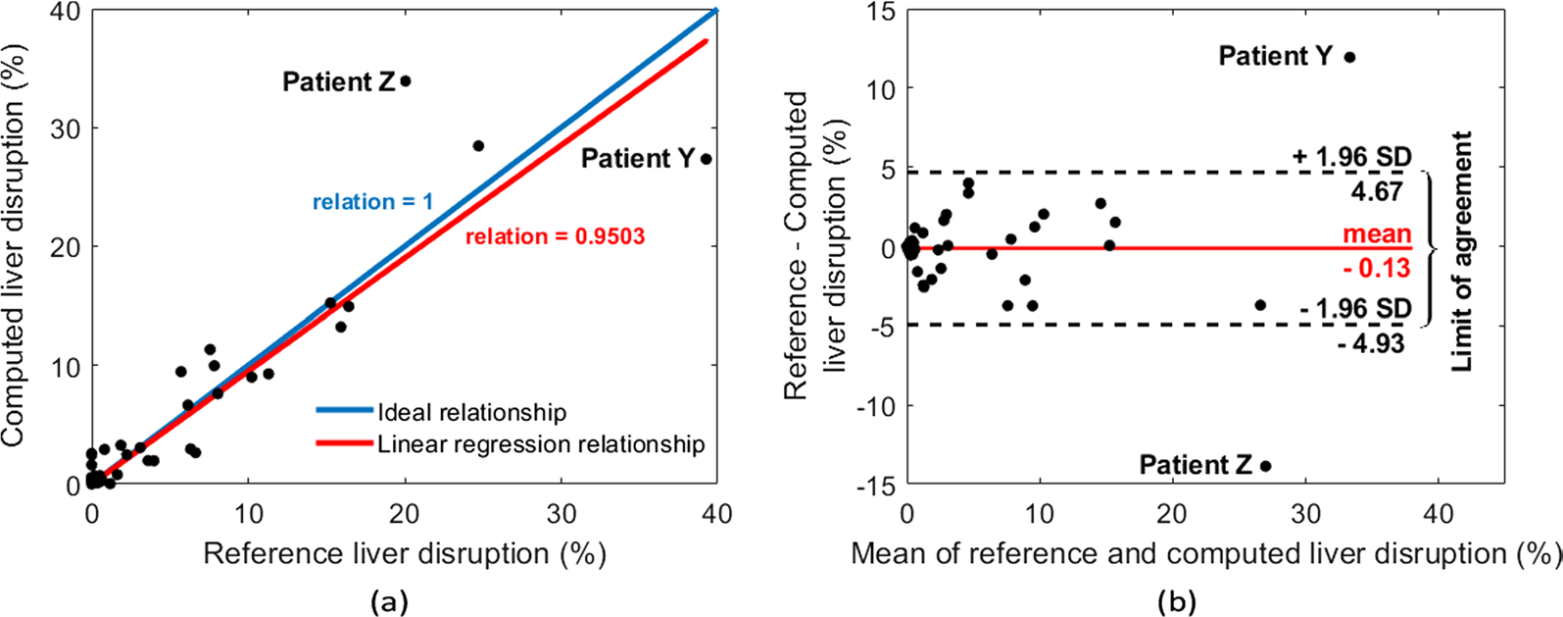
(**a**) Linear regression relation between the computed and reference LDIs. Each point corresponds to one patient. (**b**) Bland–Altman plot that indicates the normality of error

LDI measures the percentage of the liver parenchyma affected by blunt traumatic injuries. Figure 8a compares the computed versus reference LDI measures for all 77 studied patients. The linear regression relation is 0.95 with *p*-value < 0.01.

**Fig. 9:**
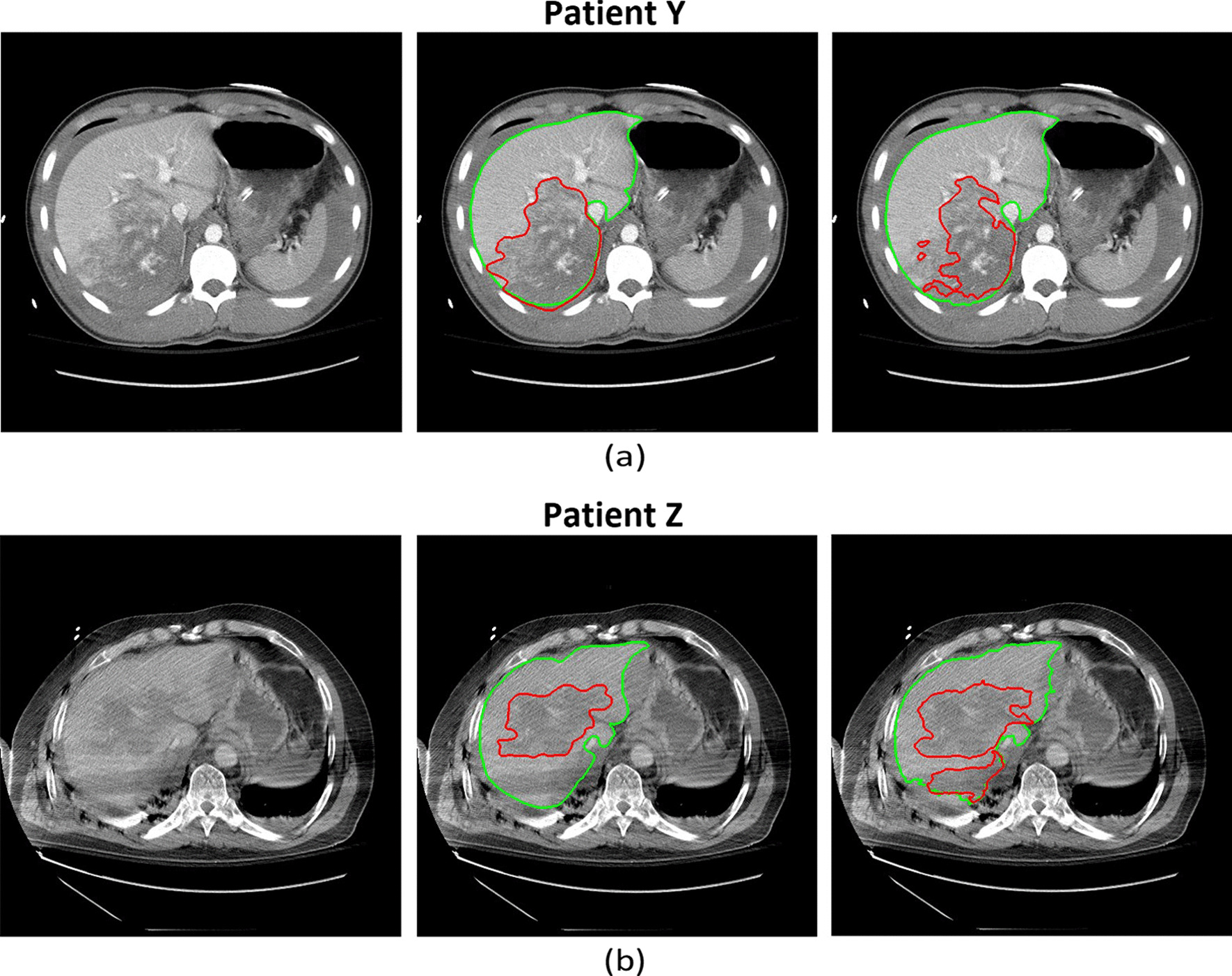
Liver trauma and parenchymal segmentation results on two patients who were determined to be outliers based on Bland–Altman analysis. (**a**) Axial contrast-enhanced CT image from Patient Y in Fig. 8. Patient Y has the largest trauma region in our dataset. (**b**) Axial contrast-enhanced CT image from Patient Z in Fig. 8. Patient Z’s CT image is distorted by linear streak artifact, which leads to a large false positive region of segmented trauma. In (**a**) and (**b**), the first column corresponds to the original CT image; the second column corresponds to the ground truth annotations; and the third column corresponds to the automated segmentation results. In both ground truth annotations and the segmentation results, the green line shows the liver contour while the red line marks the contour of trauma regions

Bland–Altman analysis was also performed to understand the potential systematic errors in computing the hepatic disruption involvement. It can be concluded from Fig. 8b that there is a negligible bias (-0.13%) with 95% confidence interval of -4.93 to 4.67%. Moreover, the Bland–Altman plot indicates two outliers that are marked as “Patient Y” and “Patient Z” in both Fig. 8a and b. Patient Y (Fig. 9a) presented with a massive liver disruption affecting 40% of the liver parenchymal, which is over 1.6 times greater than the next largest liver disruption. Since the model had not seen such a large injury in the training phase, it can be concluded that it failed to learn such a pattern, and did not segment the whole injured region. Patient Y’s recall and precision scores are, respectively, 63.68% and 92.24%, indicating the algorithm’s high performance in avoiding false positives. Patient Z’s CT scan shows a strong beam hardening artifact [22]. This streaking artifact appeared as a dark band and was misdiagnosed by the algorithm. This error might have occurred as there were no other similar cases in the training folds. These issues can potentially be addressed by extending the dataset in the future.

## Discussion

The purpose of this study is to develop an end-to-end framework that can detect and quantitatively assess the severity of liver traumas with respect to the percentage of liver parenchyma injured. For this purpose, the percentage the liver parenchyma affected by traumatic injuries was automatically computed, as it is an important component of the AAST liver injury scale. The proposed framework provides real-time quantitative information about the injury that was not accessible before due to the cumbersome manual process to annotate all images included in a 3D CT scan. As a result, we envision that this system enables objective, continuous injury severity scoring in the future to supplement the current AAST grading. Moreover, this system can be used as a triage tool by rapidly assessing liver injury and its severity as well as for monitoring volumetric progression or improvements of the trauma region at multiple time points.

The proposed algorithm employed a deep learning backbone to segment the initial liver parenchyma and trauma masks. These masks were then refined during a post-processing step by integrating human domain knowledge about the location and intensity of injury into the model. The model achieved Dice similarity coefficients of 96.13% and 51.21%, respectively, in segmenting liver and trauma regions. The Dice score for liver trauma segmentation reached 72.45% for considerable injuries with over 5% of LDI. Moreover, of the 43 non-trauma cases, 40 patients were detected to have < 1% of LDI showing high performance of the model in avoiding false positives. With regard to creating the diagnostic model, our algorithm achieved a linear regression relation of 0.95 between the computed versus reference LDI measurements. It can be concluded that the proposed algorithm can accurately quantify the extent of liver parenchymal trauma.

There is no liver trauma-specific benchmark to evaluate the liver trauma segmentation performance against; however, in an independent traumatic brain injury segmentation study [41], it was shown that there is considerable inter-physician variability in delineating brain hematoma regions. Specifically, for subtle hematoma regions with less than 25 cc of blood, it was shown that there is 50.20% agreement between two skilled radiologists in delineating the lesions as measured by the Dice similarity coefficient. This issue is due to the fact that in lesion studies, including liver hematoma detection, the border between the affected region and the adjacent healthy tissue is not necessarily well-separated.

To our knowledge, no prior study has previously described automated methods to identify and assess the severity of liver trauma using abdominopelvic CT images. The mechanism and severity of trauma can lead to significant variations in the size and shape of injured regions on CT scans. Moreover, non-traumatic pre-existing conditions, such as fatty liver and congestive hepatopathy, may significantly affect the liver parenchymal’s attenuation in CT scans. Given these sources of variation, developing a generalizable algorithm is challenging but necessary. In this work, we sought to address these challenges by integrating human domain knowledge about the location and intensity distribution of injuries into the model during the post-processing step. We also took advantage of classical image processing techniques, including 3D active contour modeling, to bring spatial coherency and intensity homogeneity to the segmented region.

The present study has a few limitations. First, despite a comprehensive process to generate the ground truth, the reference labels used to train and evaluate the models are best estimates rather than a definitive label. This is because the edges of the injury and organs are not always distinctly visible in CT images. This issue introduces not only noise into the training phase but also uncertainty into the labels against which the performance is measured that can adversely affect the performance metrics. For example, as shown in Fig. 7a and c, although the ground truth and automated segmentation results for trauma do not thoroughly overlap, the segmented regions do not visually appear to be less accurate than the ground truth. The adverse effect of these inconsequential deviations on the performance metrics is greater on smaller regions.

In addition to imperfect labels, artifacts introduced into CT scans during image collection (Fig. 9b) are another source of noise. In this study, the CT scans affected by artifacts are not excluded as long as radiologists can make a diagnosis. While the results on CT scans with strong artifacts are not perfect, excluding those scans from the study would make the study less representative of routine clinical practice. Another limitation involves the lack of enough massively injured cases (Fig. 9a) in our dataset to effectively learn their patterns. The low representation of massive cases could be because those patients went straight to surgery and did not have a CT scan prior to intervention. Further improvements can be achieved via more extensive studies in which massive trauma and non-trivial image artifacts are represented adequately.

Regarding the future direction of this investigation, other major types of such liver trauma, including subcapsular hematoma and active bleeding should also be automatically assessed to develop a comprehensive liver trauma assessment tool. Automated detection of active arterial bleeding would be a critical component of this future system as it is a severe injury that requires immediate intervention.

## Conclusion

This study is the first to automatically identify and assess liver trauma utilizing contrast-enhanced CT without taking advantage of any prior knowledge about the presence of the injury. We developed a fully automated framework capable of providing objective and quantitative information about the presence and extent of liver trauma using deep learning and image processing techniques. This model is generalizable to heterogeneous appearing livers on CT scans of patients with pre-existing liver conditions, including fatty liver and congestive hepatopathy. The accuracy of the model for both blunt trauma and non-trauma patients supports this system’s potential to enhancing the medical decision-making process.

## Supplementary Information

**Additional file 1.** U-net Architecture and Specifications.

## Data Availability

The datasets generated and/or analyzed during the current study were collected by UMHS. The University of Michigan’s Innovation Partnerships (UMIP) unit will handle potential charges/arrangements of the use of data by external entities, using such methods as material transfer agreements. The executable code developed in this work is available from the corresponding author upon UMIP approval. Please contact UMIP (innovationpartnerships@umich.edu) for data inquiries.

## Abbreviations

AAST: The American Association for the Surgery of Trauma
ACM: Active Contour Model
CT: Computed Tomography
IRB: Institutional Review Board
LDI: Liver disruption involvement
RVD: Relative Volume Difference
UMHS: University of Michigan Health
System VOE: Volumetric Overlap Error
