# Supplementary material for: A deep learning framework for automated detection and quantitative assessment of liver trauma
Source: BMC Med Imaging. 2022 Mar 8;22:39. doi: 10.1186/s12880-022-00759-9 (PMC8905785; doi:10.1186/s12880-022-00759-9)
Supplement: Supplementary file 1 — Additional file 1. U-net Architecture and Specifications. [file 12880_2022_759_MOESM1_ESM.pdf]

Supplementary Information: A Deep Learning Framework for Automated Detection and Quantitative Assessment of Liver Trauma

Supplementary Method

U-net Architecture used to generate liver parenchyma and trauma segmentation masks.

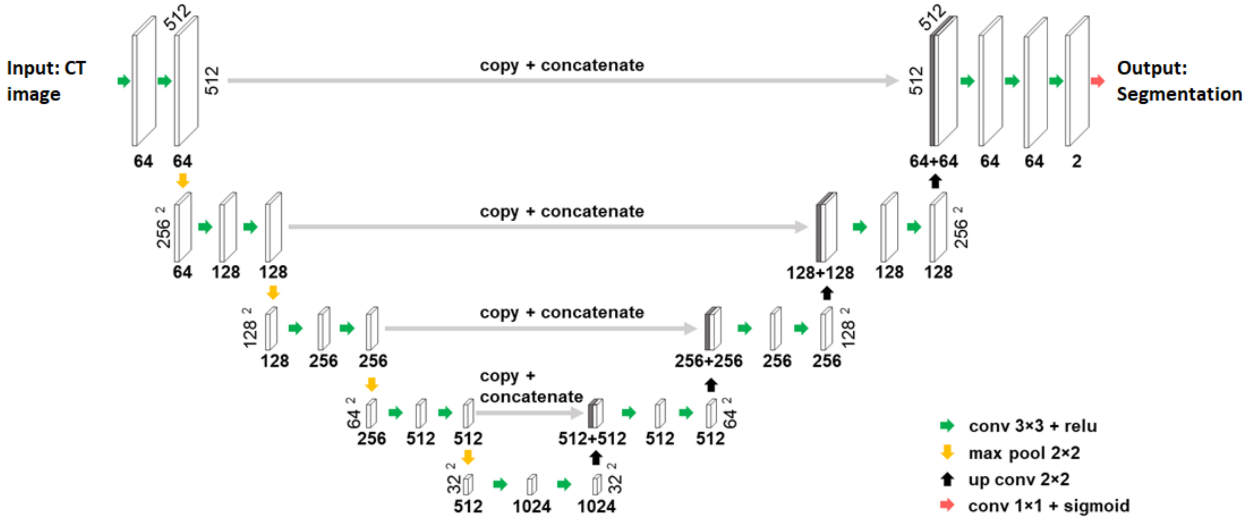

Supplementary Figure 1. Illustration of the baseline U-net architecture used for segmentation. This figure is borrowed from [41].

Liver segmentation U-net specifications. The U-net model for liver segmentation is trained for 3000 epochs, and each epoch is learned on 10 patients. An Adam optimizer with a learning rate of 2×10^{-4} is used to minimize the binary cross-entropy loss.

Liver disruption segmentation U-net specifications. The U-net model for liver disruption segmentation is trained for 4000 epochs, and each epoch is learned on 10 patients. An Adam optimizer with a learning rate of 5×10^{-5} is used to minimize the binary cross-entropy loss.
